# Cost Impact of a Novel Pre-transplant Risk Assessment Tool for Early Acute Rejection in Kidney Transplant Patients

**DOI:** 10.36469/001c.146282

**Published:** 2026-05-26

**Authors:** Jacie T. Cooper, John E. Schneider

**Affiliations:** 1 Avalon Health Economics, Miami, Florida; 2 Health Economics and Outcomes Research Avalon Health Economics

**Keywords:** risk stratification, early acute rejection, immunosuppression, kidney transplantation

## Abstract

**Background:** Accurately predicting outcomes and stratifying risks remains a challenge in kidney transplant patients. The Pre-Transplant Risk Assessment (PTRA) predicts risk of early acute rejection (EAR) in deceased donor transplant recipients, enabling clinicians to tailor immunosuppression strategies using individual immune response risk. **Objectives:** We developed a cost-impact analysis of adding PTRA to the current standard of care to predict the risk of EAR compared with the current protocol-driven immunosuppression in standard-risk kidney transplant patients. **Methods:** A decision tree model evaluated 90-day and 2-year outcomes post kidney transplantation. PTRA testing stratified patients as either high or low risk for EAR. Reduced maintenance immunosuppression protocols were modeled in low-risk PTRA patients compared with standard protocol, reducing adverse drug event readmissions, cytomegalovirus infections, graft loss, and deaths with a functioning graft. An increased maintenance immunosuppressive regimen was modelled for high-risk PTRA patients, reducing acute rejections and graft loss. **Results:** The standard-risk population cohort consisted of 71.4% low-risk PTRA and 28.6% high-risk PTRA patients. The model generated per-patient cost savings of 358inthefirst90daysposttransplantationand13 234 per-patient over 2 years. If PTRA were used in all 14 468 patients falling into the standard risk immunosuppression protocol, 191 475 915couldbesavedover2yearsatthecohortlevel.IfPTRAweretoimpactinductionimmunosuppressionprotocols,per−patientsavingswouldexceed4000 over 90 days and $27 000 over 2 years. The definitions used for risk stratification and the assumed immunosuppression protocols for patient cohorts are generalized assumptions expected to align with most clinician approaches. We assumed that the reduction on clinical outcomes would be equivalent to the overall percentage of patients with expected treatment regimen impacts from PTRA. Variations in clinical decision-making regarding treatment pathways given PTRA risk stratification will influence the potential clinical impact. **Conclusions:** Risk stratification with PTRA utilization prior to kidney transplant in the standard-risk population could generate significant cost savings in the 90-day and 2-year time periods post kidney transplantation.

## INTRODUCTION

Kidney transplantation is the leading treatment option for managing end-stage kidney disease, significantly improving survival rates, and reducing the long-term health burdens associated with dialysis.^1^ In 2023 a record 21 042 deceased donor kidney transplants (DDKT) were performed in the United States.^2^ Despite the increases in kidney transplantations, in 2020, 554 038 Americans were living with end-stage kidney disease reliant on dialysis and only 229 887 were living with a kidney transplant.^3,4^ In 2023, there were 141 886 adults on the waiting list, 10.8% of which had a previous failed transplant.

Kidney transplantation recovery generally consists of an immunosuppression regimen to limit EAR and ultimately avoid graft loss. Personalized immunosuppression regimens are essential to optimizing patient outcomes; patients should ideally receive enough immunosuppression to avoid rejection of the new kidney while avoiding over-immunosuppression that can lead to avoidable adverse events, including cardiovascular risks, infections, and malignancies.^5,6^ Unfortunately, accurately predicting outcomes and stratifying risk for EAR and transplant failure remains a significant challenge. While tools for transplant failure risk assessment such as human leukocyte antigen (HLA) mismatch analyses help assess alloimmune risk,^7^ evaluating HLA mismatches has been shown to poorly predict acute rejection.^8^ Additionally, tests for adaptive and innate immune responses, as well as non-HLA antibodies, are not yet standard practice.^7^ These limitations create barriers to implementing personalized immunosuppression strategies, highlighting the need for improvement in predictive tools.

The Pre-Transplant Risk Assessment (PTRA; One Lambda, a ThermoFisher Brand) is a pretransplantation test performed on blood that analyzes the RNA expression profile of a select gene set using a machine learning–derived algorithm to predict the risk of early acute rejection in DDKT recipients following transplantation. The test supports clinicians in tailoring immunosuppression strategies based on prognostic personalized assessment of individual immune response risk. This test can improve patient outcomes by identifying patients who are appropriate candidates for immunosuppression management changes from standard risk assessment measures. Our objective was to develop a cost-impact analysis of adding PTRA to the current standard of care to predict the risk of occurrence of early acute rejection (EAR) in the first 60 days following transplant, compared with the current protocol-driven immunosuppression in kidney transplant patients. A decision tree structure was utilized to characterize possible patient pathways in the model, which evaluated outcomes over a 2-year span post kidney transplantation.

### Adverse Outcomes

Early acute rejection remains a critical factor contributing to poor kidney transplantation outcomes. Rates of EAR range from 15% to 30% when including subclinical and borderline rejections, contributing significantly to increased cost of care, graft failure, and patient morbidity and mortality.^9,10^ The majority of acute rejections occur within the first 6 months after kidney transplantation.^11^ Delayed graft function, occurring in 5% to 50% of DDKT, can lead to even higher costs and worse graft and patient survival, highlighting the importance of early and accurate risk assessment for long-term transplant success.^12–14^

Adverse events (AEs) related to immunosuppressive therapy in kidney transplant recipients are common and can lead to significant complications, including readmissions. Approximately 28% of Medicare kidney transplantation payments are associated with 30-day readmissions, often caused by AEs.^15^ If not addressed effectively, AEs can compromise graft survival and impact patient health outcomes. EAR and AEs impose significant clinical, humanistic, and economic burden on the US healthcare system.^6,16^

Kidney graft failure can lead to significant economic burden on healthcare systems, due mainly to expensive interventions such as treatment of cell-mediated and antibody-mediated rejection, return to dialysis, retransplantation, longer hospital stays, and hospital readmissions.^17,18^ In a study analyzing 17 644 kidney transplant recipients in the United States, graft failure was estimated to lead to an incremental lifetime cost of $78 079 per patient, amounting to a total economic burden of $1.38 billion and a loss of 29 289 quality-adjusted life-years (QALYs).^19^ A major contributor to graft loss is antibody-mediated rejection (ABMR), which has been estimated to be responsible for up to 60% of late transplant failures.^20^ ABMR treatment costs are substantial, ranging from $49 000 to $155 000 per episode.^21^ ABMR commonly leads to interstitial fibrosis and tubular atrophy, leading to eventual graft failure. This requires a patient to return to dialysis, leading to added humanistic and economic burden.

Long-term immunosuppressive therapies required to prevent graft rejection can cost between $10 000 and $14 000 annually per patient.^22^ However, the cost of graft failure, estimated at $176 710, is significantly more burdensome.^18^ Annual costs of dialysis are approximated at $40 000, further emphasizing the importance of optimizing immunosuppressive therapies that contribute to long-term kidney allograft survival.^23^

### Standard of Care

The current approach to pretransplant risk assessment is highly heterogeneous among institutions, as there is not a standard risk-assessment tool for optimizing immunosuppression post transplantation. Generally, assessments rely on patient characteristics, donor kidney information, and patient/donor compatibility. However, PTRA only assesses risk based on patient-specific characteristics, so risk group definitions applied in the model are only dependent on recipient-related risk factors. A pretransplantation “high immunologic risk” patient can be defined as one with a history of a previous failed kidney transplant or with a class I or class II calculated panel-reactive antibody (cPRA) result between 80% and 100%.^24^ The “standard risk” patient is one who does not meet any of the high-risk criteria, or who was a high-risk patient with a previous malignancy for whom clinicians seek to minimize immunosuppression even in the context of high immunologic risk. Most clinicians would generally designate a “low-risk” population as one that meets all low-risk criteria (first transplant, age >60 years, and cPRA between 0% and 19%).

### Pretransplant Testing

The PTRA test examines expression levels of 29 select genes involved in T-cell signaling and activation, chemokine and complement initiation, cellular energy (ATP/ADP) functions, autophagy-lysosomal trafficking, B-cell proliferation, and NK cell activation. The expression levels of these 29 genes are analyzed using a proprietary algorithm, the output of which is a risk score between 0 and 100. The results of a prospective, international clinical study (NCT04727788) validated the PTRA test to stratify the risk of kidney allograft early acute rejection in DDKT.^25^ The PTRA test is currently approved as a laboratory developed test.

PTRA is performed on peripheral blood collected upon admission for kidney transplantation, as a tool for predicting risk of EAR in the first 2 months following transplantation. A high-risk PTRA score has the potential to identify DDKT recipients who would not be identified as high risk for EAR using standard assessments. This allows PTRA to support medical management with more careful monitoring and the potential for higher intensity immunosuppression and, in turn, lowering risk for EAR.^7^ A low-risk PTRA score identifies patients with a low probability of experiencing an early acute rejection in the first 2 months post transplant, allowing for a more personalized approach to immunosuppression management with lower doses of potentially harmful antirejection regimens. This approach can reduce the risk of serious infections, cardiovascular complications, drug toxicity, and malignancy.^6,16,26^ The PTRA high-risk score hazard ratio for EAR, 7.25 (*P* = .008), supports the ability of clinicians to stratify their patients’ risk and augment the ability to identify patients most likely to benefit from modification of immunosuppression. The PTRA test is expected to be utilized by clinicians to aid treatment decisions in patients within the standard-risk group standard of care (SOC), defined as all DDKT not meeting the predefined exclusion criteria for high or low risk.

## METHODS

### Population

The model cohort was based upon 2023 data from the Scientific Registry of Transplant Recipients (SRTR) for patients receiving DDKT. The population was further limited to patients who do not fall clearly into the low-risk or high-risk categorization when assessed for EAR with SOC protocols, where a high-risk patient can be defined as one with a history of a previous failed kidney transplant or with a class I or class II cPRA result between 80% and 100%, and a low-risk patient was one who was receiving their first transplant, was younger than 60, and had a cPRA between 0% and 19%.^24^ The standard risk patient was one who did not meet any of the low-risk or high-risk criteria, or who was a high-risk patient with a previous malignancy for whom clinicians sought to minimize immunosuppression even in the context of high immunologic risk. This represented the population for which physicians will likely implement the PTRA risk stratification tool to support decisions regarding the early course of immunosuppression management.

### Model Structure

**Figure 1.**
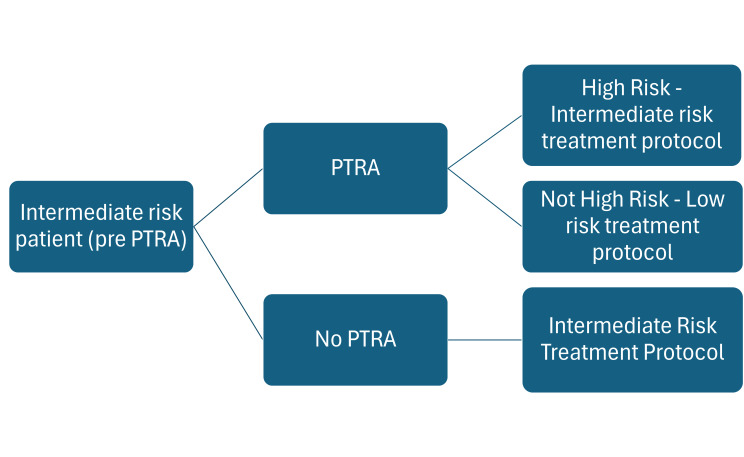
Decision Tree Structure Abbreviation: PTRA, Post-Transplant Risk Assessment.

The model encompassed a 2-year time frame after kidney transplantation. Outcomes were assessed at 90 days to derive potential savings relevant to hospitals using the diagnostic-related group payment structure, as well as at the 1-year and 2-year time points to provide insight to payers. The cost-impact model was implemented as a decision tree (**Figure 1**), which limited the SOC population most likely considered for testing with PTRA. The model compared the current standard treatment pathway (STP) protocol vs an adjusted STP guided by a PTRA test. The SOC standard-risk group followed the STP currently used in clinical practice (calcineurin inhibitors [CNIs] plus antimetabolite plus steroids).^26^ All SOC-assessed patients were assumed to follow the same maintenance immunosuppression protocol, although it is recognized that protocols may vary between centers and clinicians. Patients assessed with PTRA are categorized as either high or low risk for EAR according to test results, which could then impact their immunosuppression regimen. PTRA-identified high-risk patients are likely to receive high-intensity maintenance immunosuppression while PTRA-identified low-risk patients may possibly follow a lower intensity immunosuppression protocol.

**Standard risk (SOC):** In the clinical validation study,^25^ 98 of the 122 (80%) patients from the trial met the SOC group criteria, which comprises the intended use group for PTRA testing.^26^ In the trial, the induction regimens were as follows: rabbit antithymocyte globulin (ATG) in 64.3%, basiliximab (an IL2RA inhibitor) in 25.5%, and alemtuzumab in 9.2%; 1% of patients utilized both ATG and an IL2RA inhibitor. All patients in the trial were given prednisone as part of their induction therapy, which included a total of 500 mg to 1 g of methylprednisolone over the first 3 to 4 days post transplant. A center-specific standard taper of prednisone was used for most patients, while 25% were assumed to utilize a rapid withdrawal of prednisone by day 7. All prednisone use was assumed to be at least 5 mg daily for the entire study.

The 2023 SRTR annual report shows that tacrolimus and mycophenolate mofetil (MMF) or mycophenolic acid (Myfortic), with or without prednisone, are used as initial maintenance immunosuppression in approximately 93.5% of kidney transplant patients.^4,27^

**PTRA-stratified patients**: PTRA test results are currently utilized after the decision point for induction therapy, which is usually administered intraoperatively or immediately following kidney transplant.^28^ The base case model therefore assumed no impact on induction immunosuppression, with modeled clinical impacts driven by changes to maintenance immunosuppression protocols generally applied after hospital discharge. The model assumed a 25% reduction in maintenance immunosuppression dosing for the low-risk PTRA-identified patients compared with the utilization with SOC protocols. This was seen as a reduction of CNI trough targets and/or reduction in mycophenolic acid derivative dosing, as well as the possibility of a steroid-free or rapid steroid withdrawal protocol. For PTRA patients identified as high risk, we assumed a 5% increase in high-intensity maintenance immunosuppression utilization compared with SOC, with the likelihood of continued steroids.

### Clinical Inputs

The post-transplantation clinical outcomes of interest for this model were acute rejection, cytomegalovirus (CMV) infection, adverse drug event (ADE) hospital admissions, death-censored graft loss, and death with a functioning graft. Patients with delayed graft function (DGF) were considered independently, as these patients usually need dialysis in the first week after transplantation and experience increased healthcare expenses compared with those without DGF.^29^ DGF occurs in approximately 31% of DDKT patients post transplant.^30–33^ The model captured impacts on the duration of initial hospitalization length of stay and subsequent outpatient dialysis for the DGF population, and identified an increased ADE readmission probability for these patients.^34^ **Table 1** shows the baseline literature-based estimates representing the 90-day, 1-year, and 2-year probabilities of each clinical event in the standard-risk SOC group.

The addition of PTRA to SOC was expected to impact most of these outcomes, except acute rejection, secondary to reductions in maintenance immunosuppression dosing in the low-risk PTRA population.^35–39^ We did not expect impacts related to CMV, infections, death-censored graft loss, or death with a functioning graft to have developed within the 90-day period; these impacts were therefore conservatively omitted from the 90-day results, although the impacts were captured in the 1- and 2-year results.

**Cytomegalovirus**: CMV is one of the most common opportunistic infections following transplantation. A systematic review of CMV risk factors identified several studies that found a positive association between intensity of immunosuppression and CMV occurrence.^39–41^ CMV has a variety of negative implications for transplant patients, including increased risk of secondary life-threatening infections, mortality, acute rejection, and early graft loss. The model assessed the reduced costs associated with treating CMV infections but did not include shortened prophylaxis from 6 months to 3 months in PTRA low-risk CMV-positive recipients.

**ADE readmissions**: According to a retrospective cohort study by Arms et al, 65% of kidney transplant recipients’ hospital readmissions were attributable to an ADE. The study also concluded that readmissions due to overimmunosuppression have steadily increased over the years, triggering heightened incidence of infections, acute kidney injury, cytopenias, and gastrointestinal complications.^6^ Acute rejection alone occurs in as many as 25% of patients and leads to costs of over $28 000 per case.^42,43^ ADE readmissions are even more common in patients with DGF, experienced by 46.1% of patients compared with 31.15% of non-DGF patients.^6,34^ By reducing the immunosuppressive regimen in PTRA-identified low-risk patients, the costs associated with ADE readmissions can be significantly reduced.

**Death-censored graft loss and death with a functioning graft**: According to a study investigating the causes of kidney graft loss, 1- and 2-year death-censored graft loss probabilities are 4.30% and 1.80%, respectively.^44^ The rates for the cohort assessed in this study were verified as reasonable compared with 2020 SRTR data that show that 5% of the DDKT patients experienced graft loss in year 1 post transplantation. Death with a functioning graft occurs in 1.80% of patients at year 1 and 1.55% of patients in year 2.^45^ Cardiovascular disease (30.8%), infections (21%), and malignancy (28.3%) are the most common causes.^44^ A precise balance between under- and over-immunosuppression to minimize graft loss in kidney transplant patients is challenging. Rejection-related graft survival can be improved with higher intensity immunosuppression regimens, while a regimen too aggressive can cause acute kidney injury, chronic nephrotoxicity, cardiovascular events, malignancies, and infections leading to graft loss or mortality.^35^ Early steroid withdrawal and lower immunosuppressive regimens in patients at low risk for rejection supports that patients can maintain graft function while limiting medication-induced events that may lead to 90-day readmissions, mortality, or graft loss.^46^ Alternatively, patients with previously unidentified high-risk factors may require more intense immunosuppressive regimens to reduce the risk of rejection related graft failure.^47^

In the base case, we assumed that the occurrence of each clinical outcome would be reduced in a 1:1 relationship with the percentage of the population impacted by PTRA-directed treatment changes. As we assumed a 25% reduction in all immunosuppressive regimens, a 25% relative reduction in nonimmunologic outcomes of interest was modeled for low-risk PTRA patients compared with current SOC patients. We modeled a 5% increase in immunosuppression dosing for high-risk PTRA patients, applied to the average SOC utilization in which approximately 87% used some combination of tacrolimus or MMF. We therefore implemented a 4.23% relative reduction in acute rejection and death-censored graft loss for the PTRA high-risk population.

As patients with DGF require dialysis and experience longer initial hospitalizations than those without DGF, we predicted further value from PTRA for the DGF population. We estimated an average 2-day shorter length of stay during the initial transplant hospitalization and an average of 3 fewer required outpatient dialysis sessions for patients with a low-risk PTRA result. There is a paucity of data on running lower tacrolimus troughs for the first 2 weeks in DGF patients,^48^ but clinicians may feel more comfortable with lower tacrolimus trough targets (6-8 mg/ml), reducing renal vascular resistance, allowing for expedited recovery of the kidney and potentially lowering baseline serum creatinine levels.^49,50^

The base case estimated occurrences of impactable clinical outcomes are shown in **Table 1** for SOC patients, low-risk PTRA patients, and high-risk PTRA patients. Literature-based 90-day, 1-year, and 2-year probabilities were cited for the patients that PTRA will impact. ADE readmission probabilities differed between the DGF and non-DGF population and thus are presented separately for each.^51^ In addition to the clinical events depicted in **Table 1**, the DGF population’s added value for low-risk PTRA patients is captured economically in the following section as reductions in both initial hospitalization length of stay and outpatient dialysis duration.

**Table 1.** Clinical Inputs

**Clinical Outcome**	**Source (Reference No.)**	**SOC, %**			**Low-Risk PTRA, %**			**High-Risk PTRA, %**		
**90-⁠Day**	**Year 1**	**Year 2**	**90-⁠Day**	**Year 1**	**Year 2**	**90-⁠Day**	**Year 1**	**Year 2**		
Acute rejection	^6,42^ ^a^	15.00	26.47	0.00	15.00	26.47	0.00	14.35	25.32	0.00
CMV infection	^40^	NA	27.35	0.00	NA	20.51	0.00	NA	27.35	0.00
ADE readmission (no DGF)	^6^ ^b^	26.60	31.14	0.00	19.95	23.35	0.00	26.60	31.14	0.00
ADE readmission (DGF)	^34^	46.10	46.10	0.00	34.58	34.58	0.00	46.10	46.10	0.00
Death-censored graft loss	^44^	NA	4.30	1.80	NA	3.23	1.35	NA	4.12	1.80
Death with a functioning graft	^45^	NA	1.80	1.55	NA	1.35	1.16	NA	1.80	1.55

Abbreviations: ADE, adverse drug event; CMV, cytomegalovirus; DGF, delayed graft function; PTRA, Post-Transplant Risk Assessment; SOC, standard of care.

^a^Acute rejection rate at 90 days was estimated from various sources reporting a range of values from 8% to 27%.^42,52–56^

^b^ADE readmission rate at 90 days was calculated as the average between studies, without acute rejections.^42,52–57^

### Economic Inputs

All economic inputs are reported in 2024 US dollars and were inflated from original values as necessary using the Bureau of Labor Statistics Consumer Price Index for medical care.^58^ The costs for all events were obtained from a targeted literature search (**Table 2**).

**Table 2.** Economic Inputs

**General Inputs**	**Source (Reference No.)**	**Cost, $**
Field		
PTRA test	^59^	2650.00
Acute rejection	^43^	28 233.52
Acute rejection (90 days)	^17^	18 476.61
CMV infection	^60^	31 060.23
ADE readmission	^61^	18 403.98
ADE readmission (90 days)	^17^	10 969.03
Death-censored graft loss	^18^	176 710.25
Death with functioning graft	^19^	178 451.30
Delayed graft function	^34^	20 938.71
Dialysis (per session)	^62^	350.00
**Baseline medication costs**	**90 Days**	**Year 1^a^**
Steroids		
Methylprednisolone intraoperatively	47.38	47.38
Methylprednisolone postoperatively	118.45	118.45
Prednisone baseline taper	147.74	382.72
Prednisone rapid withdrawal	8.47	8.47
Maintenance immunosuppression		
Tacrolimus with trough goal 8-10 kg/ml^b^	3204.00	16 600.76
Mycophenolate mofetil 1 gm po BID or mycophenolate sodium (Myfortic) 360-720 mg BID	2262.67	10 989.12

Abbreviations: ADE, adverse drug event; BID, twice daily; CMV, cytomegalovirus; po, by mouth; PTRA, Post-Transplant Risk Assessment.

^a^Year 1 costs represent the average cost of a full course of treatment with each medication.

^b^Reductions in tacrolimus utilization for low-risk PTRA patients reflect a reduction in the trough goal (to 6-8 kg/ml) for the first 6 months post-transplant.

The calculations for any mg/kg dosing assumed an 80 kg patient, which is consistent with the average patient weight in the PTRA clinical validation study.^25^ Baseline dosing, tapering, utilization, and unit costs of maintenance medications are shown in **Supplementary Table S1**. All average baseline medication costs are presented in **Table 2**.

## RESULTS

In the validation study, after testing with PTRA, 71.4% of the tested SOC arm were categorized as low risk and 28.6% as high risk. All patients in the PTRA intended-use arm with a low-risk result were assumed to switch to a lower-intensity maintenance immunosuppression regimen and reduced ADE-related clinical outcomes as described above, while 5% of the high-risk PTRA patients would receive higher-intensity maintenance immunosuppression regimens and reduced rates of acute rejection and death censored graft loss. For instance, a 4.23% reduction in acute rejection for high-risk PTRA patients reduced the 1-year incidence from 26.47% to 25.32%, avoiding $28 234 per case and totaling approximately $1 342 000 in acute rejection savings for the high-risk cohort alone. The aligning clinical outcomes experienced by each population are shown in **Table 1**. The resulting average per-patient costs for 90 days, year 1, and year 2 following kidney transplantation are shown in **Table 3**. These costs were calculated by using the 2023 SRTR deceased donor population of 20 746 patients and subtracting the pediatric population and high-risk patients (retransplant, and patients with a cPRA of 80%-100%). This left a total of 14 468 patients in the standard risk SOC immunosuppression protocol.

**Table 3.** Per-Patient Costs

**Field**	**PTRA**			**SOC**		
**90 Days^a^**	**Year 1**	**Year 2**	**90 Days^a^**	**Year 1**	**Year 2**	
Global population costs, $						
PTRA test	2650.00	2650.00	NA	NA	NA	NA
Immunosuppression and steroids	18 172.93	33 710.04	19 703.98	18 961.23	38 031.56	23 577.41
Acute rejection	2737.11	7380.85	NA	2771.49	7473.58	NA
CMV infection	NA	6978.01	NA	NA	8494.97	NA
Death censored graft loss	NA	6147.38	2573.32	NA	7598.54	3180.78
Death with a functioning graft	NA	2638.53	2272.07	NA	3212.12	2766.00
Non-DGF population-specific costs (73.7%), $						
ADE readmission	2396.44	4706.85	NA	2917.40	5730.08	NA
DGF population-specific costs (26.3%)						
ADE readmission	4153.74	6969.19	NA	5056.72	8484.23	NA
Initial transplant hospitalization	4945.52	15 953.30	NA	6491.00	20 938.71	NA
Outpatient dialysis	NA	4150.00	NA	NA	4900.00	NA

Abbreviations: ADE, adverse drug event; CMV, cytomegalovirus; DGF, delayed graft function.

^a^90-day costs should not be totaled with year 1 and year 2 costs; the annual costs are already inclusive of 90-day expenses.

**Figure 2.**
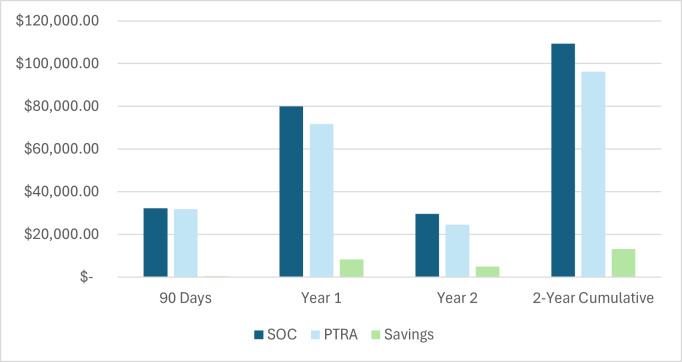
PTRA Costs and Savings Over Time vs SOC (Per Tested Patient) Abbreviations: PTRA, Post-Transplant Risk Assessment; SOC, standard of care.

Per-patient costs and savings for the 2-year period are shown graphically in **Figure 2**. The results indicate that the average SOC (standard risk) patient would cost $8259.62 less in year 1, $4974.82 in year 2, and $13 234.44 cumulatively through 2 years by implementing PTRA testing. In the first 90 days, PTRA would save $357.56 per tested patient. Utilizing PTRA testing for 14 468 patients would generate $191 475 915.21 in savings over 2 years for the entire cohort.

### Scenario Analysis

Although the PTRA test is currently administered immediately prior to transplant surgery, it is reasonable that the test may be administered earlier to enable a larger impact. To capture the implications of PTRA utilization on induction, we modeled a scenario in which PTRA is administered prior to the induction therapy decision-point (based on an 80 kg patient). Induction therapy medication costs are presented for reference in **Supplementary Table S3**.

In this scenario, we assume the following impacts on induction medications in addition to the base case impacts on maintenance immunosuppression: (1) 50% of ATG/thymoglobulin patients utilize a 4.5 mg/kg dose instead of 6 mg/kg after a low-risk PTRA result, (2) 30% of ATG/thymoglobulin patients switch to an IL2RA after a low-risk PTRA result, (3) 90% of IL2RA patients switch to ATG/thymoglobulin after a high-risk PTRA result, and (4) 10% of IL2RA patients switch to alemtuzumab after a high-risk PTRA result. With these adjustments, there is a 76.43% clinical impact for low-risk PTRA patients and a 29.85% clinical impact for high-risk PTRA patients. In this scenario, use of PTRA generates $4011.22 per patient in savings in the first 90 days, with $27 998.84 savings over the 2-year period. Complete results by cost-driver, arm, and timeframe can be found in **Supplementary Table S4**.

We also pressure-tested the model in scenario analyses for extreme variation in PTRA impact, as the actual effect from these changes in immunosuppressive regimens is unknown. We therefore incorporated a “worst case” and a “best case” scenario wherein the model applied impact at extreme values. In the worst-case scenario, the 25% reduction in immunosuppression for low-risk patients resulted in a 10% reduction in immunosuppression-related outcomes, while the risk reduction for graft loss in high-risk patients was decreased from 5% to 2.5%. In the worst-case scenario, a 40% reduction in immunosuppression-related outcomes was modeled for low-risk patients and a 7.5% reduction in graft loss was modeled for high-risk patients. In the worst-case scenario, costs would increase with PTRA by $511.60 per-patient in the first 90 days post transplantation. However, savings would be generated after 12 months, saving $4994.70 cumulatively over 2 years. Results from this scenario analysis are shown in **Supplementary Table S5**. In the best-case scenario, 90-day savings are expected to be $1226.72, and 2-year cumulative savings reach $21 474.19 per PTRA-tested patient. Expanded results for this scenario are shown in **Supplementary Table S6**.

### Sensitivity Analysis

A one-way deterministic sensitivity analysis was performed to assess the robustness of model results to reasonable variations in input parameters. To test this, we varied each parameter independently by 20% in either direction (higher and lower) and recorded the resulting 5-year cost impact results for each. The results of this analysis are displayed as tornado diagrams, with the 90-day and 2-year cumulative results depicted in **Figure 3**. The corresponding data tables are presented in **Supplementary Tables S7 and S8**. The percentage of patients receiving a low-risk PTRA result had the largest impact on overall savings at both the 90-day and 2-year time points. Ninety-day savings were also sensitive to changes in the PTRA cost, probability and cost of delayed graft function, and the probability and cost of ADE readmissions. Over the 2-year time period, the model was sensitive to the costs of MMF, tacrolimus, and the PTRA test. The analysis supports the model’s findings of significant expected cost savings from PTRA, as per-patient cost savings remain greater than $9000 in every scenario over 2 years. Only the PTRA result and PTRA cost fields negate cost savings in the 90-day period. Savings remain robust to changes in all other input parameters.

## DISCUSSION

This study estimated the value of testing SOC DDKT recipients with PTRA immediately prior to the time of kidney transplantation by tailoring immunosuppression to individual patient risk, reducing costs and a generating a reduction in clinical events. Reduction of immunosuppression in PTRA low-risk patients and increasing immunosuppression in PTRA high-risk patients was estimated to reduce hospital readmission within the first 90 days for ADEs and acute rejection, reduce the incidence of CMV infection/disease, and reduce death-censored graft loss and death with a functioning graft over the first 2 years after kidney transplantation.

Current practice guidelines have led to a significant level of overimmunosuppression, as clinicians generally resort to more intense immunosuppression in patients without distinct low-risk characteristics.^26,37^ Implementation of the PTRA test in SOC protocols may allow clinicians to more confidently tailor immunosuppression at a reduced level, enabling the decrease of medication-related adverse events and unfavorable clinical outcomes. It may also allow for more intense immunosuppression for some patients with a high-risk PTRA result, reducing acute rejection and its downstream effects. It is important to note that increased immunosuppression has a decreasing utility at a certain threshold even for high-risk patients. As immunosuppressive therapies can have significant side effects, the correct balance must be identified which maximizes organ life while minimizing ADEs.^16,63,64^

If PTRA were to be used prior to induction immunosuppression administration, it could lead to additional savings by adjusting induction therapy to lower doses of ATG or use of abciximab at a lower direct cost, and a lower risk of lymphocyte depleting antibody effects on CMV, malignancies, and a reduction in ADE related early hospital admissions.^53,65^ This may have an inverse positive effect on the high-risk patient population, ensuring that they receive the appropriate induction selection to reduce acute rejection and its downstream effects, potentially reducing the cost of treating cellular and antibody-mediated rejection, graft failure and mortality.

### Additional PTRA Value

PTRA also has the potential to impact long-term clinical outcomes that are not captured in this 2-year analysis. For example, improving renal function by reducing calcineurin-inhibitor target levels can reduce risk of cardiovascular death.^66^ Further, every 5 mL decrease in eGFR below 45 mL/min has been shown to increase the risk of cardiovascular events by 17%.^67^ Body weight, hypertension, hyperlipidemia, new-onset diabetes after transplant, and acute rejection are also risk factors for coronary heart disease, and lower graft and patient survival, all of which can be reduced with modifications to immunosuppression.^68–71^ Monitoring for de novo donor-specific antibodies and ABMR can also be quite costly, but were not specifically captured in this analysis.^72^

Conversion from CNIs to belatacept is another potential value driver for PTRA-stratified patients. Belatacept-based treatment regimens are proven to be associated with improved outcomes including reduced incidence of new-onset diabetes, lowered blood pressure, and reduced cardiovascular events.^73,74^ Having a low-risk PTRA result is likely to reduce the concern for acute rejection, potentially increasing those considered eligible for the conversion. For patients converting to belatacept, we estimate that rates of new-onset diabetes, cardiovascular events, and chronic CNI toxicity could be reduced by up to 10%.

PTRA as a tool to personalize immunosuppression may be particularly valuable in the older adult population.^75^ Older adults have a reduced immune response to transplanted organs, supporting the safety of reduced immunosuppression protocols.^76^ Elderly patients experience greater difficulties in tolerating immunosuppression in comparison to younger patients, and have increased risk of serious side effects, such as infections, cardiovascular events, and acute kidney injury, making identifying those at low risk of rejection key to reduce dosing.^77^ While PTRA is expected to improve outcomes among all standard-risk kidney transplant patients, the per-patient value described in this analysis may yet be an underestimation when looking specifically at older adults.^78^

Verici Dx has also developed Tutivia, a post-transplant peripheral blood gene expression signature to predict risk of acute rejection. Like PTRA, Tutivia identifies patients at high or low risk of acute rejection. The clinical validation study has shown a positive predictive value for acute rejection of 0.60 and a negative predictive value of 0.79, with a hazard ratio of 5.74 to predict risk acute rejection.^79^ Utilization of Tutivia in addition to pretransplant testing with PTRA may amplify the positive effects of personalized immunosuppression regimens, particularly by informing treatment decision-making through serial testing with Tutivia.

Finally, PTRA testing can assist hospitals to reach goals from the implementation of the Increasing Organ Transplant Access model being implemented by the Centers for Medicare & Medicaid Services (CMS) in July 2025.^80^ The model aims to do three things. CMS will financially incentivize participating hospitals, driven by increasing kidney transplant volumes, reducing transplant discard rates, and increasing post-transplantation survival for the first 2 years.^80^ Utilization of PTRA is expected to directly influence the quality domain by increasing post-transplant survival. The measures for each center will include all their kidney transplant patients. The performance incentive can be up to $15 000 per Medicare patient annually. Failure to meet a certain standard, compared with the other transplant centers, can result in up to a $2000 reduction in CMS reimbursement per Medicare patient.^80^

### Limitations

This study has several limitations warranting recognition. First, the definitions used for risk stratification are generalized assumptions that reflect what we expected most clinicians to consider when identifying patients of standard or high-risk for acute rejection and graft failure. We recognized that the patient and donor characteristics utilized to stratify patients vary significantly between centers and between clinicians. However, we expected the simplistic stratification utilized in the model to be representative of general SOC protocols. Similarly, we assumed general immunosuppression protocols for high and standard risk patient cohorts. While most of this utilization is supported by literature, the dosing and specific drug selections will vary between transplant centers and clinicians.

While most of the clinical inputs data relied on data specific to the DDKT population, some clinical outcomes data were unavailable for DDKT alone and were therefore reflective of the general kidney transplantation population. However, as most kidney transplantations conducted (~72%) are from deceased donors, the data utilized remain adequate.^81^ Further, DDKT patients are expected to have worse clinical outcomes than living donor recipients, so any skewing of input data would constitute a conservative base value for the DDKT population.^33^

Assumptions were made regarding the impact of PTRA on clinical outcomes due to a lack of data. In the absence of outcomes data after PTRA use, we assumed that the reduction on clinical outcomes would be equivalent to the overall percentage of patients with expected treatment regimen impacts from PTRA. Variations in clinical decision-making regarding treatment pathways given PTRA risk stratification will influence the potential clinical impact. Despite this uncertainty, we could see from the sensitivity analysis that the clinical savings are robust to variation in the level of clinical impact triggered by PTRA. Finally, this analysis did not incorporate the sensitivity and specificity of PTRA into the calculations. To adequately incorporate test accuracy into future models, a study needs to be conducted wherein patients follow PTRA-driven immunosuppression protocols and clinical outcomes (ie, graft failure occurrence) are assessed. A budget impact model is also warranted to incorporate the impact of utilization uptake.

## CONCLUSIONS

This analysis quantified the expected value of PTRA risk-stratification in DDKT patients at the time of kidney transplantation. Tailoring immunosuppression regimens to patient risk enables significant cost savings related to ADE-driven outcomes, as well as a reduction in undesired complications. Reducing the degree of maintenance immunosuppression for PTRA-identified low-risk patients reduces avoidable complications from immunosuppressive treatment. Increasing maintenance immunosuppression for high-risk PTRA patients enables reduction in rates of acute rejection and the downstream effects related to de novo deterministic sensitivity analysis formation, antibody-mediated rejection, and death censored graft loss. If the timing of PTRA testing was adjusted to enable induction immunosuppression regimen impacts, we would expect savings to increase more than twofold. Outcomes remained robust to reasonable variations of input parameter values at all time points.

### Disclosures

J.T.C. is an employee of Avalon Health Economics, where J.E.S. is the CEO and principal. Avalon Health Economics was compensated for the completion of this work.

## Supplementary Material

Online Supplementary Material

## References

[1] Wang J. H., Hart A. (2021). Global perspective on kidney transplantation: United States. Kidney 360.

[2] (2024). SRTR Data 2023.

[3] Johansen K. L., Chertow G. M., Foley R. N.. (2021). US Renal Data System 2020 Annual Data Report: Epidemiology of Kidney Disease in the United States. Am J Kidney Dis.

[4] Lentine K. L., Smith J. M., Miller J. M.. (2025). OPTN/SRTR 2023 Annual Data Report: Kidney. Am J Transplant.

[5] Bisognano J., Schneider J. E., Davies S.. (2021). Cost-impact analysis of baroreflex activation therapy in chronic heart failure patients in the United States. BMC Cardiovasc Disord.

[6] Arms M. A., Fleming J., Sangani D. B., Nadig S. N., McGillicuddy J. W., Taber D. J. (2018). Incidence and impact of adverse drug events contributing to hospital readmissions in kidney transplant recipients. Surgery.

[7] Bestard O., Thaunat O., Bellini M. I.. (2022). Alloimmune risk stratification for kidney transplant rejection. Transplant Int.

[8] Do Nguyen H. T., Wong G., Chapman J. R.. (2016). The association between broad antigen HLA mismatches, eplet HLA mismatches and acute rejection after kidney transplantation. Transplant Direct.

[9] Oweira H., Ramouz A., Ghamarnejad O.. (2022). Risk factors of rejection in renal transplant recipients: a narrative review. J Clin Med.

[10] Rampersad C., Balshaw R., Gibson I. W.. (2022). The negative impact of T cell-mediated rejection on renal allograft survival in the modern era. Am J Transplant.

[11] Longer-term risks of a kidney transplant.

[12] Wu W. K., Famure O., Li Y., Kim S. J. (2015). Delayed graft function and the risk of acute rejection in the modern era of kidney transplantation. Kidney Int.

[13] Siedlecki A., Irish W., Brennan D.C. (2011). Delayed graft function in the kidney transplant. Am J Transplant.

[14] Yarlagadda S. G., Coca S. G., Formica R. N., Jr., Poggio E. D., Parikh C. R. (2009). Association between delayed graft function and allograft and patient survival: a systematic review and meta-analysis. Nephrol Dial Transplant.

[15] Forster A. J., Murff H. J., Peterson J. F., Gandhi T. K., Bates D. W. (2003). The incidence and severity of adverse events affecting patients after discharge from the hospital. Ann Intern Med.

[16] Rostaing L., Jouve T., Terrec F., Malvezzi P., Noble J. (2023). Adverse drug events after kidney transplantation. J Pers Med.

[17] Famure O., Kim E. D., Au M.. (2021). What are the burden, causes, and costs of early hospital readmissions after kidney transplantation?. Prog Transplant.

[18] Cooper M., Schnitzler M., Nilubol C., Wang W., Wu Z., Nordyke R. J. (2022). Costs in the year following deceased donor kidney transplantation: relationships with renal function and graft failure. Transpl Int.

[19] Sussell J., Silverstein A. R., Goutam P.. (2020). The economic burden of kidney graft failure in the United States. Am J Transplant.

[20] Baldwin W. M., 3d, Valujskikh A., Fairchild R. L. (2016). Mechanisms of antibody-mediated acute and chronic rejection of kidney allografts. Curr Opin Organ Transplant.

[21] Muduma G., Odeyemi I., Smith-Palmer J., Pollock R. F. (2016). Review of the clinical and economic burden of antibody-mediated rejection in renal transplant recipients. Adv Ther.

[22] James A., Mannon R. B. (2015). The cost of transplant immunosuppressant therapy: is this sustainable?. Curr Transplant Rep.

[23] Pockros B. M., Finch D. J., Weiner D. E. (2021). Dialysis and total health care costs in the united states and worldwide: the financial impact of a single-payer dominant system in the US. J Am Soc Nephrol.

[24] Stratta R. J., Rohr M. S., Sundberg A. K.. (2004). Increased kidney transplantation utilizing expanded criteria deceased organ donors with results comparable to standard criteria donor transplant. Ann Surg.

[25] Concepcion B. P., Bestard O., Samaniego-Picota M.. A Pre-Kidney Transplant Blood-Based Next-Generation Sequencing Assay to Predict Early Acute Rejection. Kidney360.

[26] Kalluri H. V., Hardinger K. L. (2012). Current state of renal transplant immunosuppression: present and future. World J Transplant.

[27] Tess M., Lapp K., Joelle N., Anne K., Charles H., Suverta B. (2023). Evaluation of a weight-based mycophenolate mofetil dosing protocol for kidney transplant maintenance immunosuppression. J Clin Nephrol.

[28] Salvadori M., Tsalouchos A. (2020). Immunosuppression in kidney transplantation. OBM Transplant.

[29] Wavamunno M. D., O'Connell P. J., Floege J., Johnson R. J., Feehally J. (2010). Comprehensive Clinical Nephrology.

[30] Bae S., Garonzik Wang J. M., Massie A. B.. (2020). Early steroid withdrawal in deceased-donor kidney transplant recipients with delayed graft function. J Am Soc Nephrol.

[31] Mannon R. B. (2018). Delayed graft function: the AKI of kidney transplantation. Nephron.

[32] Wu W.K., Famure O., Li Y., Kim S.J. (2015). Delayed graft function and the risk of acute rejection in the modern era of kidney transplantation. Kidney Int.

[33] Zens T. J., Danobeitia J. S., Leverson G.. (2018). The impact of kidney donor profile index on delayed graft function and transplant outcomes: a single-center analysis. Clin Transplant.

[34] Kim D. W., Tsapepas D., King K. L.. (2020). Financial impact of delayed graft function in kidney transplantation. Clin Transplant.

[35] Pilch N. A., Bowman L. J., Taber D. J. (2021). Immunosuppression trends in solid organ transplantation: the future of individualization, monitoring, and management. Pharmacotherapy.

[36] Cheungpasitporn W., Lentine K. L., Tan J. C.. (2021). Immunosuppression considerations for older kidney transplant recipients. Curr Transplant Rep.

[37] Wojciechowski D., Wiseman A. (2021). Long-term immunosuppression management: opportunities and uncertainties. Clin J Am Soc Nephrol.

[38] Sanders-Pinheiro H., da Silveira S. T., Carminatti M.. (2012). Excessive immunosuppression in kidney transplant patients: prevalence and outcomes. Transplant Proc.

[39] Soliman K., Calimlim I. K., Perry A.. (2024). Evolving trends in immunosuppression use and cytomegalovirus infection risk over the past decade in kidney transplantation. Transpl Infect Dis.

[40] Raval A. D., Kistler K. D., Tang Y., Murata Y., Snydman D. R. (2021). Epidemiology, risk factors, and outcomes associated with cytomegalovirus in adult kidney transplant recipients: a systematic literature review of real-world evidence. Transplant Infect Dis.

[41] Kotton C. N., Kumar D., Caliendo A. M.. (2018). The Third International Consensus Guidelines on the Management of Cytomegalovirus in Solid-organ Transplantation. Transplantation.

[42] Bloom R. D., Bromberg J. S., Poggio E. D.. (2017). Cell-free DNA and active rejection in kidney allografts. J Am Soc Nephrol.

[43] First M. R., Lee D., Lewis P., Rose S. (2017). An economic analysis of the cost effectiveness of blood gene expression profiling in kidney transplant recipients. J Health Med Econ.

[44] Mayrdorfer M., Liefeldt L., Wu K.. (2021). Exploring the complexity of death-censored kidney allograft failure. J Am Soc Nephrol.

[45] Ng M. S. Y., Jones A. T., Mallett A. J., O'Shaughnessy M. M. (2024). Better kidney allograft survival despite higher-risk donor and recipient characteristics between 1995 and 2014. Nephrol Dial Transplant.

[46] Phanish M. K., Hull R. P., Andrews P. A., Popoola J., Kingdon E. J., MacPhee I. A. M. (2020). Immunological risk stratification and tailored minimisation of immunosuppression in renal transplant recipients. BMC Nephrol.

[47] Jones-Hughes T., Snowsill T., Haasova M.. (2016). Immunosuppressive therapy for kidney transplantation in adults: a systematic review and economic model. Health Technol Assess.

[48] Liu Y., Liu H., Shen Y., Chen Y., Cheng Y. (2018). Delayed initiation of tacrolimus is safe and effective in renal transplant recipients with delayed and slow graft function. Transplant Proc.

[49] Curtis J., Dubovsky E., Whelchel J., Luke R., Diethelm A., Jones P. (1986). Cyclosporin in therapeutic doses increases renal allograft vascular resistance. Lancet.

[50] Naesens M., Kuypers D. R., Sarwal M. (2009). Calcineurin inhibitor nephrotoxicity. Clin J Am Soc Nephrol.

[51] Kawakita S., Beaumont J. L., Jucaud V., Everly M. J. (2020). Personalized prediction of delayed graft function for recipients of deceased donor kidney transplants with machine learning. Sci Rep.

[52] Harhay M., Lin E., Pai A.. (2013). Early rehospitalization after kidney transplantation: assessing preventability and prognosis. Am J Transplant.

[53] Luan F. L., Barrantes F., Roth R. S., Samaniego M. (2014). Early hospital readmissions post-kidney transplantation are associated with inferior clinical outcomes. Clin Transplant.

[54] Nguyen M. C., Avila C. L., Brock G. N.. (2020). "Early" and "late" hospital readmissions in the first year after kidney transplant at a single center. Clin Transplant.

[55] Huang E., Sethi S., Peng A.. (2019). Early clinical experience using donor-derived cell-free DNA to detect rejection in kidney transplant recipients. Am J Transplant.

[56] Ganji M. R., Broumand B. (2007). Acute cellular rejection. Iran J Kidney Dis.

[57] Hogan J., Arenson M. D., Adhikary S. M.. (2019). Assessing predictors of early and late hospital readmission after kidney transplantation. Transplant Direct.

[58] Consumer Price Index for All Urban Consumers (CPI-U). CUUR0000SAM.

[59] Thermo Fisher Data on file; 2025..

[60] Cheng W. Y., Avery R. K., Thompson-Leduc P.. (2022). Evaluation of treatment patterns, healthcare resource utilization, and costs among patients receiving treatment for cytomegalovirus following allogeneic hematopoietic cell or solid organ transplantation. J Med Econ.

[61] McAdams-Demarco M. A., Grams M. E., Hall E. C., Coresh J., Segev D. L. (2012). Early hospital readmission after kidney transplantation: patient and center-level associations. Am J Transplant.

[62] Wei S. (2024). Dialysis cost with and without insurance in 2024.

[63] Claeys E., Vermeire K. (2019). Immunosuppressive drugs in organ transplantation to prevent allograft rejection: mode of action and side effects. J Immunol Sci.

[64] Manickavasagar R., Thuraisingham R. (2020). Post renal-transplant malignancy surveillance. Clin Med (Lond).

[65] Padiyar A., Augustine J. J., Hricik D. E. (2009). Induction antibody therapy in kidney transplantation. Am J Kidney Dis.

[66] Meier-Kriesche H. U., Baliga R., Kaplan B. (2003). Decreased renal function is a strong risk factor for cardiovascular death after renal transplantation. Transplantation.

[67] Weiner D. E., Carpenter M. A., Levey A. S.. (2012). Kidney function and risk of cardiovascular disease and mortality in kidney transplant recipients: the FAVORIT trial. Am J Transplant.

[68] Birdwell K. A., Park M. (2021). Post-Transplant cardiovascular disease. Clin J Am Soc Nephrol.

[69] Israni A.K., Snyder J.J., Skeans M.A.. (2010). Predicting coronary heart disease after kidney transplantation: Patient Outcomes in Renal Transplantation (PORT) Study. Am J Transplant.

[70] Sharif A., Chakkera H., de Vries A. P. J.. (2024). International consensus on post-transplantation diabetes mellitus. Nephrol Dial Transplant.

[71] Palepu S., Prasad G. V. (2015). New-onset diabetes mellitus after kidney transplantation: current status and future directions. World J Diabetes.

[72] Hart A., Zaun D., Itzler R., Schladt D., Israni A., Kasiske B. (2021). Cost, healthcare utilization, and outcomes of antibody-mediated rejection in kidney transplant recipients in the US. J Med Econ.

[73] Vanrenterghem Y., Bresnahan B., Campistol J.. (2011). Belatacept-based regimens are associated with improved cardiovascular and metabolic risk factors compared with cyclosporine in kidney transplant recipients (BENEFIT and BENEFIT-EXT studies). Transplantation.

[74] Terrec F., Jouve T., Naciri-Bennani H.. (2020). Late conversion from calcineurin inhibitors to belatacept in kidney-transplant recipients has a significant beneficial impact on glycemic parameters. Transplant Direct.

[75] Meier-Kriesche H.-U., Kaplan B. (2001). Immunosuppression in elderly renal transplant recipients. Drugs Aging.

[76] Peeters L. E. J., Andrews L. M., Hesselink D. A., de Winter B. C. M., van Gelder T. (2018). Personalized immunosuppression in elderly renal transplant recipients. Pharmacol Res.

[77] Khan S., Loi V., Rosner M. H. (2017). Drug-induced kidney injury in the elderly. Drugs Aging.

[78] Haugen C. E., King E. A., Bae S.. (2018). Early hospital readmission in older and younger kidney transplant recipients. Am J Nephrol.

[79] Bestard O., Augustine J., Wee A.. (2024). Prospective observational study to validate a next-generation sequencing blood RNA signature to predict early kidney transplant rejection. Am J Transplant.

[80] Centers for Medicare & Medicaid Services Increasing Organ Transplant Access (IOTA) Model.

[81] Mohan S., Yu M., Maclay L. M.. (2025). Outcomes for patients with a deceased donor kidney offer in the new allocation system. Kidney Int Rep.

