# Supplementary material for: Cost Impact of a Novel Pre-transplant Risk Assessment Tool for Early Acute Rejection in Kidney Transplant Patients
Source: J Health Econ Outcomes Res. 2026 May 26;13(1):146282. doi: 10.36469/001c.146282 (PMC13218370; doi:10.36469/001c.146282)
Supplement: Online Supplementary Material [file jheor_2026_13_1_146282_346178.pdf]

Journal of Health Economics and Outcomes Research

Online Supplementary Material

Cost Impact of a Novel Pre-transplant Risk Assessment Tool for Early Acute Rejection in Kidney Transplant Patients. *JHEOR*. 2026;13(1):189-199. doi:10.36469/jheor.2026.146282

Table S1: Medication Unit Costs

Table S2: Medication Utilization by Arm

Table S3: Induction Immunosuppression Costs

Table S4: Per-Patient Costs (Induction Immunosuppression Scenario Analysis)

Table S5: Per-Patient Costs (Worst Case Scenario Analysis)

Table S6: Per-Patient Costs (Best Case Scenario Analysis)

Table S7: One-Way Sensitivity Analysis Data (90-Day)

Table S8: One-Way Sensitivity Analysis Data (2-Year)

This supplementary material has been provided by the authors to give readers additional information about their work.

This is an open-access article distributed under the terms of the Creative Commons Attribution 4.0 International License (CCBY-4.0). View this license's legal deed at http://creativecommons.org/licenses/by/4.0 and legal code at http://creativecommons.org/licenses/by/4.0/legalcode for more information.

Table S1. Medication Unit Costs

	Vials	Cost Per Vial, $	Average Cost, $	References
Induction				
Thymoglobulin, 6 mg/kg	20[a]	940.00	18 800.00	
Thymoglobulin, 4.5 mg/kg	15[b]	940.00	14 100.00	
Campath, 20-30 mg	2.5	0.00	0.00	NA
Basiliximab, 20 mg IV	2	3244.57	6489.14	1
Solu-Medrol, 500 mg intraoperatively	1	47.38	47.38	2
Solu-Medrol, 2-3 doses postoperatively	2.5	47.38	118.45	2
Maintenance				
Tacrolimus with trough goal, 8-10 kg/ml	12	1068.00	16 600.76	1
MMF, 1 g po BID	12	1094.00	17 004.90	1
Myfortic, 360-720 mg BID	6	828.89	4973.34	
Prednisone (baseline taper) from 1 mg/kg/day to 0.4 mg/kg/day at 1 month and 0.15mg/kg/day at 1 year[c]	8473.45	0.05	382.72	3
Prednisone (rapid withdrawal), 1 mg/kg/day day 1, 0.5 mg/kg/day days 2 and 3, 0.25 mg/kg/day days 4 and 5	187.50	0.05	8.47	3
Prednisone (steroid-free)	0	0.00	NA	NA

Abbreviations: BID, twice daily; MMF, mycophenolate mofetil; twice daily; po, by mouth; PTRA, Post-Transplant Risk Assessment.
[a]An 80 kg patient would require 120 mg (5 units of 25 mg vials) per dose for 4 doses, totaling 20 vials.
[b]An 80 kg patient would require 120 mg (5 units of 25 mg vials) per dose for 3 doses, totaling 15 vials.
[c]Baseline prednisone taper informed by Serrano et al.[4]

Table S2. Medication Utilization by Arm

Medication	Utilization[a]		
	Standard Risk (SOC), %	Low-Risk PTRA, %	High-Risk PTRA, %
ATG/thymoglobulin, 6 mg	64.29	64.29	64.29
ATG/thymoglobulin, 4.5 mg	0.00	0.00	0.00
IL2RA (baziliximab)	25.51	27.14	0.00
Campath 1H	9.18	8.57	10.71
ATG/thymoglobulin and IL2RA	1.02	0.00	3.57
Solu-Medrol intraoperatively	100.00	100.00	100.00
Solu-Medrol postoperatively	100.00	100.00	100.00
Baseline taper	75.00	50.00	90.00
Rapid withdrawal	25.00	50.00	10.00
Steroid-free	0.00	0.00	0.00
Tacrolimus with trough goal 8-10 kg/ml	80.00	60.00	84.00
MMF, 1 g po BID/Myfortic, 360 mg to 720 mg BID2	93.70	70.28	98.39

Abbreviations: ATG, antithymocyte globulin; BID, twice daily; MMF, mycophenolate mofetil; twice daily; po, by mouth; PTRA, Post-Transplant Risk Assessment; SOC, standard of care.
[a]Utilization of induction immunosuppression medications informed by data from the PTRA validation study (not yet published). Utilization for PTRA low and high risk calculated using approach described in manuscript. Steroid utilization assumed. Tacrolimus SOC utilization informed by literature.[5]
[b]MMF and Myfortic listed as one line item as patients will either use one or the other, not both. Utilization of MMF or Myfortic informed by SRTR data.[6]

Table S3. Induction Immunosuppression Costs

Induction Immunosuppression	Cost, $
ATG/thymoglobulin, 6 mg/kg total dose	18 800.00
ATG/thymoglobulin, 4.5 mg/kg total dose	14 100.00
IL2RA (basiliximab)	6489.14
Alemtuzumab[a]	0.00
ATG/thymoglobulin and IL2RA	25 289.14

[a]Alemtuzumab is currently supplied by the manufacturer at no cost.

Table S4. Per-Patient Costs (Induction Immunosuppression Scenario Analysis)

Field	PTRA, $			SOC, $		
	**90 Days**	**Year 1**	**Year 2**	**90 Days**	**Year 1**	**Year 2**
Global population costs						
PTRA test	2650.00	2650.00	NA	NA	NA	NA
Immunosuppression and steroids	16 036.59	33 101.58	19 703.98	18 961.23	38 031.56	23 577.41
Acute rejection	2535.10	6836.13	NA	2771.49	7473.58	NA
CMV infection	NA	4637.56	NA	NA	8494.97	NA
Death-censored graft loss	NA	3500.08	1465.15	NA	7598.54	3180.78
Death with a functioning graft	NA	1753.56	1510.01	NA	3212.12	2766.00
Non-DGF population-specific costs (73.7%)						
ADE readmission	1324.74	2601.92	NA	2917.40	5730.08	NA
DGF population-specific costs (26.3%)						
ADE readmission	2296.17	3852.54	NA	5056.72	8484.23	NA
Initial hospitalization dialysis	4945.52	15 953.30	NA	5506.88	20 938.71	NA
Outpatient dialysis	NA	4150.00	NA	NA	4900.00	NA

Abbreviations: ADE, adverse drug event; CMV, cytomegalovirus; DGF, delayed graft function; NA, not applicable; PTRA, Post-Transplant Risk Assessment; SOC, standard of care.

Table S5. Per-Patient Costs (Worst Case Scenario Analysis)

Field	PTRA, $			SOC, $		
	**90 Days**	**Year 1**	**Year 2**	**90 Days**	**Year 1**	**Year 2**
Global population costs						
PTRA test	2650.00	2650.00	NA	NA	NA	NA
Immunosuppression and steroids	18 641.26	36 067.78	22 061.72	18 961.23	38 031.56	23 577.41
Acute rejection	2754.30	7427.22	NA	2771.49	7473.58	NA
CMV infection	NA	7888.19	NA	NA	8494.97	NA
Death-censored graft loss	NA	7008.65	2933.85	NA	7598.54	3180.78
Death with a functioning graft	NA	2982.69	2568.42	NA	3212.12	2766.00
Non-DGF population-specific costs (73.7%)						
ADE readmission	2709.02	5320.79	NA	2917.40	5730.08	NA
DGF population-specific costs (26.3%)						
ADE readmission	4695.53	7878.22	NA	5056.72	8484.23	NA
Initial hospitalization dialysis	4945.52	15 953.30	NA	5506.88	20 938.71	NA
Outpatient dialysis	NA	4150.00	NA	NA	4900.00	NA

Abbreviations: ADE, adverse drug event; CMV, cytomegalovirus; DGF, delayed graft function; NA, not applicable; PTRA, Post-Transplant Risk Assessment; SOC, standard of care.

Table S6. Per-Patient Costs (Best Case Scenario Analysis)

Field	PTRA, $			SOC, $		
	90 Days	Year 1	Year 2	90 Days	Year 1	Year 2
Global population costs						
PTRA test	2650.00	2650.00	NA	NA	NA	NA
Immunosuppression and steroids	17 704.60	31 352.30	17 346.24	18 961.23	38 031.56	23 577.41
Acute rejection	2719.91	7334.49	NA	2771.49	7473.58	NA
CMV infection	NA	6067.84	NA	NA	8494.97	NA
Death censored graft loss	NA	5286.11	2212.79	NA	7598.54	3180.78
Death with a functioning graft	NA	2294.37	1975.71	NA	3212.12	266.00
Non-DGF population-specific costs (73.7%)						
ADE readmission	2083.86	4092.91	NA	2917.40	5730.08	NA
DGF population-specific costs (26.3%)						
ADE readmission	3611.95	6060.17	NA	5056.72	8484.23	NA
Initial hospitalization dialysis	4945.52	15 953.30	NA	5506.88	20 938.71	NA
Outpatient dialysis	NA	4150.00	NA	NA	4900.00	NA

Abbreviations: ADE, adverse drug event; CMV, cytomegalovirus; DGF, delayed graft function; NA, not applicable; PTRA, Post-Transplant Risk Assessment; SOC, standard of care.

Table S7. One-Way Sensitivity Analysis Data (90-Day)

Parameter	Base Value		20% Lower		20% Higher	
	Input	Cost Impact	Input	Cost Impact	Input	Cost Impact
Dialysis session cost, $	350.00	$357.56	280.00	$357.56	420.00	$357.56
Dialysis sessions avoided with low-risk PTRA, n	3.00	$357.56	2.40	$357.56	3.60	$357.56
Death with a functioning graft, % (1-year)	1.80	$357.56	1.44	$357.56	2.16	$357.56
Death with a functioning graft, % (2-year)	1.55	$357.56	1.24	$357.56	1.86	$357.56
Death censored graft loss, % (2-year)	1.80	$357.56	1.44	$357.56	2.16	$357.56
Death with a functioning graft cost, $	178 451.30	$357.56	142 761.04	$357.56	214 141.56	$357.56
Death censored graft loss, % (1-year)	4.30	$357.56	3.44	$357.56	5.16	$357.56
Total CMV population, %	27.35	$357.56	21.88	$357.56	32.82	$357.56
CMV infection cost, $	31 060.23	$357.56	24 848.19	$357.56	37 272.28	$357.56
Graft loss cost, $	176 710.25	$357.56	141 368.20	$357.56	212 052.30	$357.56
IL2RA (baziliximab) cost, $ (90 days)	6489.14	$357.56	5191.31	$357.56	7786.97	$357.56
ATG/thymoglobulin 6 mg cost, $ (90 days)	18 800.00	$357.56	15 040.00	$357.56	22 560.00	$357.56
ATG/thymoglobulin 4.5 mg cost, $ (90 days)	14 100.00	$357.56	11 280.00	$357.56	16 920.00	$357.56
Rapid steroid withdrawal cost, $ (90 days)	187.50	$357.56	150.00	$357.79	225.00	$357.33
Baseline steroids taper cost, $ (90 days)	3271.02	$357.56	2616.82	$353.55	3925.23	$361.57
Acute rejection, % (90 days)	15.00	$357.56	12.00	$350.68	18.00	$364.43
Acute rejection cost, $ (90 days)	18 476.61	$357.56	14 781.29	$350.68	22171.93	$364.43
Myfortic 360mg to 720 mg BID cost, $ (90 days)	1243.34	$357.56	994.67	$338.42	1492.00	$376.70
MMF 1 g po BID cost, $ (90 days)	3282.00	$357.56	2625.60	$307.04	3938.40	$408.08
DGF patients with an ADE readmission, % (90 days)	46.10	$357.56	36.88	$301.57	55.32	$413.54
Probability of ADE-related readmission after transplant, % (90 days)	26.60	$357.56	21.28	$285.66	31.92	$429.45
Tacrolimus cost, $ (90 days)	3204.00	$357.56	2563.20	$273.34	3844.80	$441.78
ADE readmission cost, $ (90 days)	10 969.03	$357.56	8775.23	$229.68	13 162.84	$485.44
Excess cost of delayed graft function, $	20 938.71	$357.56	16 750.97	$48.46	25 126.45	$666.65

Table S7. One-Way Sensitivity Analysis Data (90-Day)

Parameter	Base Value		20% Lower		20% Higher	
	Input	Cost Impact	Input	Cost Impact	Input	Cost Impact
DGF hospital cost reduction with low risk PTRA, %	33.33	$357.56	26.67	$48.46	40.00	$666.65
Delayed graft function, %	31.00	$357.56	24.80	$24.78	37.20	$690.34
PTRA cost, $	2650.00	$357.56	2120.00	$887.56	3180.00	($172.44)
Low risk PTRA, %	71.43	$357.56	57.14	($270.90)	85.71	$986.01

Abbreviations: ADE, adverse drug event; BID, twice daily; CMV, cytomegalovirus; DGF, delayed graft function; MMF, mycophenolate mofetil; po, by mouth; PTRA, Post-Transplant Risk Assessment.

Table S8. One-Way Sensitivity Analysis Data (2-Year)

Parameter	Base Value		20% Lower		20% Higher	
	Input	Cost Impact	Input	Cost Impact	Input	Cost Impact
Solu-Medrol intra-op cost, $	47.38	$13 234.44	37.90	$13 234.44	56.86	$13 234.44
Solu-Medrol post-op cost, $	118.45	$13 234.44	94.76	$13 234.44	142.14	$13 234.44
ATG/thymoglobulin 6 mg cost, $	14 100.00	$13 234.44	11280.00	$13 234.44	16 920.00	$13 234.44
ATG/thymoglobulin 4.5 mg cost, $	18 800.00	$13 234.44	15040.00	$13 234.44	22 560.00	$13 234.44
Rapid steroid withdrawal cost, $	8.47	$13 234.44	6.78	$13 234.67	10.16	$13 234.21
Baseline steroids taper cost, $	382.72	$13 234.44	306.17	$13 224.05	459.26	$13 244.83
Acute rejection, %	26.47	$13 234.44	21.18	$13 215.90	31.76	$13 252.99
Acute rejection cost, $	28 233.52	$13 234.44	22586.82	$13 215.90	33 880.22	$13 252.99
Dialysis session cost, $	350.00	$13 234.44	280.00	$13 187.94	420.00	$13 280.94
Dialysis sessions avoided with low-risk PTRA, n	3.00	$13 234.44	2.40	$13 187.94	3.60	$13 280.94
IL2RA (baziliximab) cost, $	6489.14	$13 234.44	5191.31	$13 154.98	7786.97	$13 313.90
Death with a functioning graft, % (1-year)	1.80	$13 234.44	1.44	$13 148.40	2.16	$13 320.48
DGF patients with an ADE readmission, %	46.10	$13 234.44	36.88	$13 140.51	55.32	$13 328.38
Death with a functioning graft, % (2-year)	1.55	$13 234.44	1.24	$13 135.66	1.86	$13 333.23
Death censored graft loss, % (2-year)	1.80	$13 234.44	1.44	$13 112.95	2.16	$13 355.93
Probability of ADE-related readmission after transplant, %	31.14	$13 234.44	24.91	$13 093.24	37.36	$13 375.65
Myfortic 360 mg to 720 mg BID cost, $	4973.34	$13 234.44	3978.67	$13 081.33	5968.01	$13 387.56
Death with a functioning graft cost, $	178 451.30	$13 234.44	142 761.04	$13 020.94	214 141.56	$13 447.95
Death censored graft loss, % (1-year)	4.30	$13 234.44	3.44	$13 016.77	5.16	$13 452.12
ADE readmission cost, $	18 403.98	$13 234.44	14 723.18	$12 999.30	22 084.78	$13 469.58
Total CMV population, %	27.35	$13 234.44	21.88	$12 931.05	32.82	$13 537.83
CMV infection cost, $	31 060.23	$13 234.44	24 848.19	$12 931.05	37 272.28	$13 537.83
Excess cost of delayed graft function, $	20 938.71	$13 234.44	16 750.97	$12 925.35	25 126.45	$13 543.54
DGF hospital cost reduction with low-risk PTRA, %	33.33	$13 234.44	26.67	$12 925.35	40.00	$13 543.54
Delayed graft function, %	31.00	$13 234.44	24.80	$12 848.35	37.20	$13 620.53
Graft loss cost, $	176 710.25	$13 234.44	141 368.20	$12 822.72	212 052.30	$13 646.17
MMF 1 g po BID cost, $	17 004.90	$13 234.44	13 603.92	$12 710.91	20 405.88	$13 757.97
PTRA cost, $	2650.00	$13 234.44	2120.00	$13 764.44	3180.00	$12 704.44

Abbreviations: ADE, adverse drug event; BID, twice daily; CMV, cytomegalovirus; DGF, delayed graft function; MMF, mycophenolate mofetil; po, by mouth; PTRA, Post-Transplant Risk Assessment.

REFERENCES

1. James A, Mannon RB. The cost of transplant immunosuppressant therapy: is this sustainable? *Current Transplant Rep*. 2015;2:113-121.
2. Pfizer. SOLU-MEDROL® (methylprednisolone sodium succinate). Information for Connecticut Prescribers of Prescription Drugs. 2024.
3. GoodRx. Prednisone 2025. Available from: https://www.goodrx.com/prednisone?label_override=prednisone&form=tablet&dosage=1mg&quantity=120&drugId=547
4. Serrano OK, Kandaswamy R, Gillingham K, et al. Rapid discontinuation of prednisone in kidney transplant recipients: 15-year outcomes from the University of Minnesota. *Transplantation*. 2017;101(10):2590-2598.
5. Israni AK, Riad SM, Leduc R, et al. Tacrolimus trough levels after month 3 as a predictor of acute rejection following kidney transplantation: a lesson learned from DeKAF Genomics. *Transpl Int.* 2013;26(10):982-989.
6. SRTR Data 2023. In: SRTR, ed. *Scientific Registry of Transplant Recipients.* 2024.
